# Evaluation of a recombinant human gelatin as a substitute for a hydrolyzed porcine gelatin in a refrigerator-stable Oka/Merck live varicella vaccine

**DOI:** 10.1186/1476-8518-5-4

**Published:** 2007-02-23

**Authors:** Vladimir Liska, Stacey A Bigert, Philip S Bennett, David Olsen, Robert Chang, Carl J Burke

**Affiliations:** 1 Vaccine Clinical Research, Merck Research Laboratories, P.O. Box 1000, UG3CD28, North Wales, PA 19454, USA; 2 Biologics and Vaccines, Merck Research Laboratories, West Point, PA 19486, USA; 3 NonClinical Statistics, Merck Research Laboratories, West Point, PA 19486, USA; 4 FibroGen, Inc., South San Francisco, CA 94080, USA

## Abstract

**Background:**

The labile nature of live, attenuated varicella-zoster virus (Oka/Merck) requires robust stabilization during virus bulk preparation and vaccine manufacturing in order to preserve potency through storage and administration. One stabilizing ingredient used in a varicella-zoster virus (VZV) vaccine is hydrolyzed porcine gelatin which represents the major protein/peptide-based excipient in the vaccine formulation.

**Methods:**

In this comparative study, a recombinant human gelatin fragment (8.5 kD) was assessed as a potential replacement for hydrolyzed porcine gelatin in an experimental live, attenuated VZV (Oka/Merck) vaccine. VZV (Oka/Merck) was harvested in two formulations prepared with either a hydrolyzed porcine gelatin or a recombinant human gelatin. Moreover, the viral stability in the experimental VZV (Oka/Merck) vaccines was evaluated under accelerated and real-time conditions in a comparative study.

**Results and discussion:**

The stabilizing effect of recombinant human gelatin on VZV (Oka/Merck) potency change during vaccine lyophilization was similar to the experimental vaccine containing porcine-derived gelatin. Vaccine viral potency changes were comparable in stabilized VZV (Oka/Merck) formulations containing either hydrolyzed porcine gelatin or recombinant human gelatin. No statistically significant difference in potency stability was observed between the vaccine formulations stored at any of the temperatures tested.

**Conclusion:**

The recombinant human gelatin demonstrated similar ability to stabilize the live attenuated VZV (Oka/Merck) in an experimental, refrigerator-stable varicella vaccine when compared to the vaccine preparation formulated with hydrolyzed porcine gelatin used in currently marketed varicella vaccine.

## Background

Varicella virus vaccine live is a lyophilized preparation of live, attenuated VZV (Oka/Merck) [1]. The inherent lability of the live varicella virus (Oka/Merck) presents a formulation challenge in terms of stabilizing and preserving vaccine viability during manufacturing, storage and administration [2]. The refrigerator-stable varicella vaccine formulation contains stabilizers such as sucrose, hydrolyzed porcine gelatin, phosphate, glutamate, and urea, as well as a live attenuated varicella virus (Oka/Merck) and residual components of MRC-5 cells [1]. Hydrolyzed porcine gelatin is a major protein/peptide-based component of the final formulation, as well as a component used in the processing of VZV (Oka/Merck) bulk intermediate [2]. The exact mechanism of gelatin-mediated protection to the vaccine virus is unknown. It is believed that gelatin provides non-covalent and non-specific protective binding to the virus particles that enhances their stability. In addition, hydrolyzed gelatin creates and maintains desirable structure/appearance of a lyophilized vaccine cake [2]. The current manufacturing process of hydrolyzed porcine gelatin yields preparations which consist of a mixture of protein fragments of different sizes [3]. Hydrolysis converts high molecular weight gelatin (>100,000 Da) to low molecular weight gelatin (between 2000 and 5000 Da) [4]. Low molecular weight gelatin is less likely to stimulate gelatin-specific IgE than high molecular weight gelatin in vaccinated subjects [5]. Currently, the incidence of anaphylactic reactions to the hydrolyzed porcine gelatin is very low (approximately 1 case per 2 million doses) [4]. In contrast, use of non-hydrolyzed gelatin in vaccine formulations by Japanese vaccine makers in the past led to higher incidence of gelatin-specific immediate-type hypersensitivity reactions in vaccinated subjects in Japan [6-10].

The implementation of alternative, well-defined substitutes for biological materials of human or animal origin in vaccine formulations is a desirable trend in pharmaceutical industry. To support this goal, recombinant human gelatin, termed FG-5001, was obtained using a yeast expression system and a completely defined fermentation and purification process (FibroGen, Inc., South San Francisco, CA). FG-5001 is a low molecular weight human sequence-based gelatin fragment (8.5 kDa) that can be used as a substitute for animal-derived material and has been shown to function as an effective alternative stabilizing ingredient in a live attenuated influenza vaccine [11].

In this study, FG-5001 was evaluated as a potential replacement for hydrolyzed porcine gelatin in an experimental refrigerator-stable varicella vaccine formulation. VZV (Oka/Merck) was harvested in two formulations prepared with either a hydrolyzed porcine gelatin or FG-5001. The stabilizing effect of FG-5001 on VZV (Oka/Merck) during vaccine lyophilization was assessed. Moreover, the short-term, as well as long-term VZV (Oka/Merck) potency stability under accelerated and real-time storage conditions was evaluated in a comparative study. VZV (Oka/Merck) potency change after a short-term stability study under accelerated conditions (37°C for 7 days) was similar for both vaccine preparations. Even more importantly, vaccine virus potency losses associated with a long-term storage under accelerated conditions at 15°C for 12 months and real-time conditions at -15°C and at 2–8°C for 24 months were similar for both hydrolyzed porcine gelatin- and recombinant human gelatin-stabilized vaccines. Thus, recombinant human gelatin, FG-5001, demonstrated a similar ability to stabilize the live attenuated VZV (Oka/Merck) in an experimental refrigerator-stable varicella vaccine when compared to the vaccine preparation formulated with a hydrolyzed porcine gelatin.

## Methods

### Preparation of experimental varicella (Oka/Merck) viral bulks

Culture flasks with VZV (Oka/Merck)-infected MRC5 cells were obtained from Merck Manufacturing Division (MMD, West Point, PA). The VZV (Oka/Merck) containing MRC5 cells were harvested into two formulations prepared with either a hydrolyzed porcine gelatin (SOL-U-PRO; Dynagel Inc., IL), or 8.5 kD recombinant human gelatin (FG-5001; Lot # 04AE001, FibroGen, Inc., CA), and further harvested in a small-scale process closely mimicking current manufacturing procedure for VZV (Oka/Merck) bulk preparation. Both processed bulks were aliquoted, placed in a liquid nitrogen batch freezer (Kwik-Freeze Freezing System, AIRCO, NJ, USA), frozen and transferred to -70°C. A set of small frozen liquid sample aliquots (1.0 mL) was submitted for VZV plaque assay analysis to determine VZV (Oka/Merck) potency changes during bulk processing for both, SOL-U-PRO- and FG-5001-containing, varicella bulks.

### Preparation of experimental, refrigerator-stable varicella vaccine samples

Varicella virus vaccine live (Oka/Merck) is a lyophilized preparation. When this refrigerator-stable vaccine is reconstituted as directed, each 0.5 mL dose contains the following: a minimum of 1350 plaque forming units (PFU) of Oka/Merck varicella virus, approximately 18 mg of sucrose, 8.9 mg of hydrolyzed gelatin, 3.6 mg of urea, 2.3 mg of sodium chloride, 0.36 mg of monosodium L-glutamate, 0.33 mg of sodium phosphate basic, 57 mcg of potassium phosphate monobasic, 57 mcg of potassium chloride. The product also contains residual components of MRC-5 cells including DNA, protein and trace quantities of neomycin and bovine calf serum from MRC-5 culture media. The product contains no preservative [1].

Experimental, refrigerator-stable varicella vaccine samples containing either the porcine hydrolyzed gelatin, or recombinant human gelatin, were prepared in a small-scale procedure closely mimicking current manufacturing process for varicella vaccine. Briefly, aliquots (40 mL) of SOL-U-PRO- and FG-5001-stabilized VZV (Oka/Merck) bulks were quickly thawed in water bath (30°C), and then diluted into their respective gelatin-containing formulations to a target potency of ≈ 4.4 log_10 _pfu/mL. Final formulated bulk (FFB) aliquots (0.7 mL) of both experimental vaccines were filled in glass vials, partially stoppered, placed in a liquid nitrogen batch freezer and frozen. These frozen FFB samples were divided into two groups. The first group was transferred to a -70°C freezer and was later used as a control to determine the VZV (Oka/Merck) potency change after lyophilization. The second group of samples was transported to the lyophilization chamber (Usifroid Lyophilizer Model SMH 101, Usifroid SA, France), and lyophilized. After lyophilization, the vaccine vials were inspected, sealed and placed in stability stations.

### Short term and long-term vaccine stability study under accelerated and real-time conditions

In addition to storage at -15°C and 2–8°C to examine real-time conditions, the stability stations used for storage were tempered at 15°C and 37°C to examine the vaccine potency stability under accelerated conditions. At pre-determined time points (37°C for 7 days; 15°C for 3, 6, 9, 12 months; 2–8°C and 15°C for 3, 6, 9, 12, 24 months) vaccine vials were removed from stability stations and stored at -70°C until submission for VZV (Oka/Merck) potency analysis. Sample vaccine vials from individual time points were analyzed together with their respective control samples which had been stored at -70°C. In addition, three sample vials from each time point were also submitted for moisture analysis from the long-term stability study executed at 2–8°C for 24 months.

### VZV (Oka/Merck) plaque assay analysis

VZV (Oka/Merck) potency in both viral bulk preparations and experimental vaccine samples were determined by VZV plaque assay with liquid overlay medium [12]. Analyzed samples (thawed liquid bulk samples and FFB liquid vaccine samples, as well as reconstituted lyophilized vaccine samples) were diluted with the stabilizer and submitted for analysis in 1 × 12 assay format (one sample in each of 12 independent assay runs). VZV (Oka/Merck) potency was defined as a log_10 _of VZV plaque forming units (PFU) per mL.

### Moisture content analysis of lyophilized vaccine samples

The amount of moisture in the lyophilized vaccine samples was determined by the Karl Fischer coulometric titration method [13] using an Aquatest™ coulometric moisture titration system (Photovolt Instruments, Inc., Minneapolis, MN) according to the manufacturer's instructions. For each analysis, average moisture content (%) was calculated based on valid results from three tested vaccine samples.

### Statistical analysis

The potency losses associated with lyophilization were calculated as the average of the differences observed between the liquid samples and the -70°C (lyophilized) samples tested in the same 12 assay runs. The standard error of the loss estimate was simply the standard deviation of the observed differences divided by the square root of the number of runs in which a difference was calculated. The same calculations were performed with the stability data for 37°C for one week. Within run differences between the -70°C (lyophilized) samples and the 37°C samples were determined and averaged across 12 independent runs. The data generated for long term stability estimation consisted of concurrent testing of "incubated" samples (those stored at -15°C, 2–8°C, and 15°C for pre-specified interval) along with control samples from the same lot which were stored only at -70°C. For each stability interval, 12 incubated vials and 12 control vials were tested, one vial each, in 12 independent assay runs. The potency loss at that interval was calculated as the average difference between the control and incubated sample within each run. This format helps to minimize the potential for run-to-run differences in the assay affecting the stability estimation. For each of the two formulations, linear regression analysis was performed using the individual loss estimates at each long term storage temperature.

## Results

### VZV (Oka/Merck) potency changes after lyophilization of experimental varicella vaccines

VZV-infected MRC5 cells were harvested into two stabilizers containing either SOL-U-PRO or FG-5001, in a small-scale process which closely mimicked the manufacturing procedure for VZV (Oka/Merck) bulks. Both, SOL-U-PRO and FG-5001-stabilized VZV (Oka/Merck) bulks were further used in the preparation of experimental refrigerator-stable varicella vaccines. These experimental varicella vaccine formulations were prepared in a small-scale formulation, filling, freezing, and lyophilization procedure closely mimicking the current manufacturing process for varicella vaccine. The VZV (Oka/Merck) potency losses associated with lyophilization were similar for both experimental, hydrolyzed porcine gelatin-(0.79 log_10 _PFU with a standard error ± 0.03) and the recombinant human gelatin-containing (0.70 log_10 _PFU with a standard error ± 0.06) varicella vaccines. After lyophilization, the vials with varicella vaccine samples were placed in the stability stations for short-term, as well as long-term varicella vaccine potency stability studies.

### Short-term thermal stability study of experimental varicella vaccines under accelerated conditions

The main objective of this experiment was to assess the effect of a recombinant human gelatin, FG-5001, on the short-term stability of VZV (Oka/Merck) potency in experimental, refrigerator-stable varicella vaccine formulation under accelerated conditions (37°C for 7 days) in a comparative study with a vaccine formulated with SOL-U-PRO. Similar potency changes were observed for both vaccine formulations, containing either porcine gelatin (0.47 ± 0.03 log_10 _PFU per 7 days) or recombinant human gelatin (0.44 ± 0.07 log_10 _PFU per 7 days), after short-term exposure to thermal stress at 37°C for 7 days. Thus, the replacement of porcine gelatin with recombinant human gelatin-based product does not appear to have a significant effect (p = 0.49) on thermal stability of VZV (Oka/Merck) as seen in this study. Moreover, lyophilized varicella vaccine formulations made with both gelatin preparations demonstrated a high percentage of cakes with excellent integrity that was maintained even after short-term exposure to thermal stress (data not shown).

### Long-term stability study under accelerated and real-time conditions

**Figure 1:**
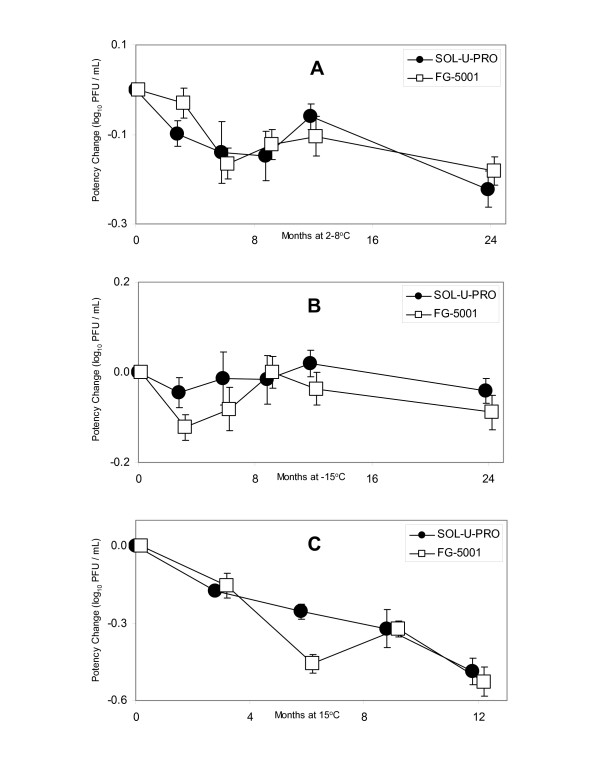
The long-term stability of VZV (Oka/Merck) formulated in hydrolyzed porcine gelatin (SOL-U-PRO), or recombinant human gelatin (FG-5001) stabilized varicella vaccine matrices. The VZV (Oka/Merck) potency change was determined as a difference between the potency of control samples stored at -70°C, and samples stored at 2–8°C (Figure 1A), and -15°C (Figure 1B) for 24 months, and 15°C (Figure 1C) for 12 months, respectively. The value of 0 for the potency loss (change) at time interval 0 months represents the stability model starting at that point. Vaccine samples were analyzed by the VZV plaque assay in format 1 × 12. The VZV potency change is in log_10 _PFU/mL.

Following lyophilization, vaccine samples were placed in stability chambers tempered at 2–8°C and -15°C for long-term (24 months) stability study under real-time conditions. In order to assess VZV (Oka/Merck) potency stability under accelerated conditions, a set of both types of vaccine samples were also placed in a stability chamber tempered at 15°C for 12 months. The virus potency losses associated with long-term storage (log10 PFU loss per month, linear regression model) at 2–8°C (Fig. 1A), -15°C (Fig. 1B), and 15°C (Fig. 1C), were similar for the two hydrolyzed porcine gelatin- and recombinant human gelatin-containing varicella vaccines (p = 0.94, 0.87, and 0.97, respectively). The loss rate estimates for each type of gelatin-stabilized vaccine, as well as a pooled estimate combining the data from both vaccines are listed in Table 1. No statistically significant difference in potency stability was observed between the vaccine formulations stored at any of the temperatures tested. During the long-term study (24 months) under real-time conditions (2–8°C), the averaged moisture content values of hydrolyzed porcine gelatin-(2.33% ± 0.12 standard error) and recombinant human gelatin-containing (2.27% ± 0.07 std error) vaccine samples were comparable (p = 0.68). No statistically significant trend in moisture content over time at 2–8°C was found for either formulation (p > 0.05).

**Table 1:** The combined potency loss rate estimates of VZV (Oka/Merck) in both, hydrolyzed porcine gelatin (SOL-U-PRO), as well as recombinant human gelatin (FG-5001) stabilized, experimental refrigerator-stable varicella vaccine formulations stored at -15°C and 2–8°C for 24 months, and at 15°C for 12 months, respectively. The potency loss rates are in log_10 _PFU per month.

	Potency Loss Rate Estimates (95%CI)		
	
Storage Temperature	SOL-U-PRO	FG-5001	Combined
-15°C	0.000 (-0.004,0.005)	0.000 (0.005, 0.005)	0.000 (-0.003, 0.004)
2–8°C	0.005 (0.000, 0.010)	0.005 (0.000, 0.010)	0.005 (0.001, 0.009)
15°C	0.033 (0.017, 0.049)	0.033 (0.017, 0.048)	0.033 (0.022, 0.044)

## Discussion

Experiments summarized above analyzed the suitability of recombinant human gelatin, FG-5001, as a replacement for hydrolyzed porcine gelatin, SOL-U-PRO, in an experimental, refrigerator-stable varicella vaccine preparation. In our study, vaccine preparations containing either SOL-U-PRO or FG-5001 demonstrated comparable VZV (Oka/Merck) short-term, as well as long-term potency stability under accelerated and real-time conditions. Statistical analysis of VZV (Oka/Merck) potency changes during the long-term stability study under real-time conditions showed no statistically significant difference in VZV potency stability for either formulation stored at any of the temperatures tested. In addition, FG-5001 performed similarly to SOL-U-PRO when it was used as a component in a process to generate bulk virus, as well as during preparation of a liquid vaccine formulation, filling into vials, freezing, and lyophilization.

In another short-term stability study under accelerated conditions, Olsen et al. [11] demonstrated that FG-5001 functioned as an effective live virus stabilizer, maintaining the titer of a live attenuated influenza strain A/Sydney CAZ-002 as well as a gelatin hydrolysate (Kind & Knox Corporation, Sioux City, IA). This study indicated the single polypeptide contained the full biological activity of a commercially available processed animal gelatin product.

Hydrolyzed animal-derived gelatins are widely used in the pharmaceutical industry as stabilizers in vaccines and other biopharmaceuticals. The heterogeneous nature of these protein mixtures creates a challenge with respect to their analytical characterization. The yeast-produced recombinant human gelatin fragment, FG-5001, is a product of defined molecular weight and physical-chemical properties, and represents a new biomaterial not previously available from animal sources [3].

While gelatin producers and end-users have investigated a number of natural and synthetic substitutes for the animal-source gelatin currently available, a universal substitute has not yet been found. On the contrary, recombinant yeast technology can provide suitable human gelatin-based materials that can be highly purified and fully characterized. These genetically distinct molecules can potentially be used as an alternative substitute for hydrolyzed animal-derived gelatins and other excipients currently used in a variety of pharmaceutical products. Even more importantly, this new technology allows the production of recombinant human-based gelatins with pre-defined molecular weight, isoelectric point (pI), guaranteed lot-to-lot reproducibility, and the ability to tailor the molecule to match a specific pharmaceutical application.
